# Consensus Statement on Proton Therapy for Prostate Cancer

**DOI:** 10.14338/IJPT-20-00031.1

**Published:** 2021-04-12

**Authors:** Curtis M. Bryant, Randal H. Henderson, R. Charles Nichols, William M. Mendenhall, Bradford S. Hoppe, Carlos E. Vargas, Thomas B. Daniels, C. Richard Choo, Rahul R. Parikh, Huan Giap, Jerry D. Slater, Neha Vapiwala, William Barrett, Akash Nanda, Mark V. Mishra, Seungtaek Choi, Jay J. Liao, Nancy P. Mendenhall, the Genitourinary Subcommittee of the Particle Therapy Co-Operative Group

**Affiliations:** 1 Department of Radiation Oncology, University of Florida College of Medicine, Gainesville, FL, USA; 2 Department of Radiation Oncology, Mayo Clinic, Jacksonville, FL, USA; 3 Department of Radiation Oncology, Mayo Clinic, Phoenix, AZ, USA; 4 Department of Radiation Oncology, Mayo Clinic Rochester, Rochester, MN, USA; 5 Department of Radiation Oncology, Rutgers Cancer Institute of New Jersey, New Brunswick, NJ, USA; 6 Department of Radiation Oncology, University of Miami Sylvester Comprehensive Cancer Center, Miami, FL, USA; 7 Department of Radiation Oncology, Loma Linda University, Loma Linda, CA, USA; 8 Department of Radiation Oncology, University of Pennsylvania, Philadelphia, PA, USA; 9 Department of Radiation Oncology, University of Cincinnati, Cincinnati, OH, USA; 10 Department of Radiation Oncology, Orlando Health, Orlando, FL, USA; 11 Department of Radiation Oncology, University of Maryland School of Medicine, Baltimore, MD, USA; 12 Department of Radiation Oncology, MD Anderson Cancer Center, Houston, TX, USA; 13 Department of Radiation Oncology, University of Washington Medical Center, Seattle, WA, USA

**Keywords:** prostate cancer, radiation therapy, proton therapy, particle therapy

## Abstract

Proton therapy is a promising but controversial treatment in the management of prostate cancer. Despite its dosimetric advantages when compared with photon radiation therapy, its increased cost to patients and insurers has raised questions regarding its value. Multiple prospective and retrospective studies have been published documenting the efficacy and safety of proton therapy for patients with localized prostate cancer and for patients requiring adjuvant or salvage pelvic radiation after surgery. The Particle Therapy Co-Operative Group (PTCOG) Genitourinary Subcommittee intends to address current proton therapy indications, advantages, disadvantages, and cost effectiveness. We will also discuss the current landscape of clinical trials. This consensus report can be used to guide clinical practice and research directions.

## Introduction

Proton therapy, a type of therapeutic radiation, features charged particles with physical properties that inherently reduce the amount of excess radiation delivered to patients when compared with photon radiation therapy. Protons deposit most of their dose at a narrow depth relative to their energy, which results in a dose distribution known as the Bragg peak. Because the entrance dose of a proton beam is relatively low and because there is minimal exit dose beyond the Bragg peak, proton therapy reduces the amount of radiation therapy absorbed by a patient's healthy tissues along the entrance and exit path of the radiation beam compared with that of photon radiation therapy. Such a reduction in excess radiation dose delivered to the bladder, rectum, bowel, and penile bulb may, in turn, reduce the risk for gastrointestinal (GI), genitourinary (GU), and sexual toxicities among patients treated for prostate cancer [1–3]. The lower integral dose of proton therapy may also reduce the risk of developing a second cancer when compared with photon-based radiation therapy [4]. Because of the relative biologic effectiveness (RBE) of proton therapy, it is predicted to be at least as effective in treating cancer as photon-based radiation therapy. Theoretically, delivering larger daily doses and fewer treatment fractions with hypofractionation may further take advantage of proton therapy's dosimetric strengths.

Proton therapy is controversial in the management of prostate cancer because of its increased cost to patients and insurers when compared with photon radiation therapy and given the current paucity of high-level evidence showing its relative benefits in improving patient quality of life (QOL), reducing toxicity, and improving cure rates among patients treated with radiation therapy. Several prospective clinical trials and retrospective outcome reviews have been published documenting the effectiveness of proton therapy in patients with prostate cancer. In this article, the GU Subcommittee of the Particle Therapy Cooperative Group (PTCOG) will summarize the results of these studies to clarify the current evidence for proton therapy's potential advantages, disadvantages, and indications. In addition, this article will summarize recent cost-effectiveness analyses comparing proton to photon radiation and provide a summary of current clinical trials analyzing proton therapy in the management of prostate cancer.

## Potential Advantages of Proton Therapy

The relative advantages and disadvantages of proton therapy when compared with photon radiation have evolved over time as the radiation oncology community continues to refine the planning and delivery techniques for both modalities. The results of those comparisons depend on the delivery technique: intensity-modulated proton therapy (IMPT) or double-scattered proton therapy versus static intensity-modulated radiation therapy (IMRT) or volumetric arc photon therapy. The outcomes and costs are further modified by the facility-specific image guidance, motion management, and robust optimization. Nevertheless, some broad conclusions can be drawn concerning the advantages of protons over photons in radiation delivery.

First, proton therapy reduces the radiation dose to organs at risk surrounding the prostate, including the bladder, bowel, and rectum, especially in the low to moderate dose range (1-50 Gy), as shown in the **Figure**. Trofimov et al [2] compared double-scattered proton therapy, IMPT, and 3-dimensional (3D) conformal photon-based radiation therapy among patients treated for prostate cancer. Radiation therapy plans for 10 patients receiving 79.2 Gy (or GyRBE) delivered at 1.8 Gy (or GyRBE) per fraction to the prostate alone were developed and compared. The authors [2] found that proton therapy significantly reduced the volume receiving 30 GyRBE (V30) to the rectum and bladder when compared with IMRT. Specifically, rectal V30 was reduced by 26% and bladder V30 was reduced by 20% with proton therapy. Rectal V50 was also reduced significantly by proton therapy, but the benefit was smaller (28% versus 34%; *P* = .027) than with lower isodoses. Vargas et al [3] compared IMRT and proton therapy using pencil-beam scanning (PBS) when delivering prostate-only radiation therapy to 78 Gy (or GyRBE) delivered at 2 Gy (GyRBE) per fraction. Plans for 10 patients were created and clinical target volume (CTV) to planning target volume (PTV) expansions were 5 mm in the axial dimension and 8 mm in the superior to inferior dimension. The authors [3] reported that proton therapy significantly reduced all rectal volumes receiving doses between 10 and 80 Gy (V10 to V80 GyRBE) when compared with IMRT among patients treated for prostate cancer. Additionally, all bladder volumes receiving between 10 to 35 Gy (V10 and V35 GyRBE) were reduced significantly by proton therapy. Similarly, Chera et al [1] compared IMRT and double-scattered proton therapy plans for a patient with high-risk prostate cancer who required pelvic nodal irradiation. Radiation therapy plans for 15 patients were created and CTVs included pelvic nodal volumes and prostate and proximal seminal vesicles. Sequential plans were delivered with the prostate and proximal seminal vesicle CTV and nodal CTV volumes treated to 46 Gy (or GyRBE) at 2 Gy (GyRBE). A boost was provided to the prostate and proximal seminal vesicle CTV volumes of 32 Gy (or GyRBE) to a total dose of 78 Gy (or GyRBE). The passively scattered proton therapy plans significantly reduced the rectal volume receiving 5 to 40 Gy (V5 to V40 GyRBE) by 53% to 71% (*P* < .05) when compared to IMRT. Proton therapy also reduced the V5 and V40 GyRBE of excess radiation delivered to the bladder by 40% to 63% (*P* < .05).

**Figure.**
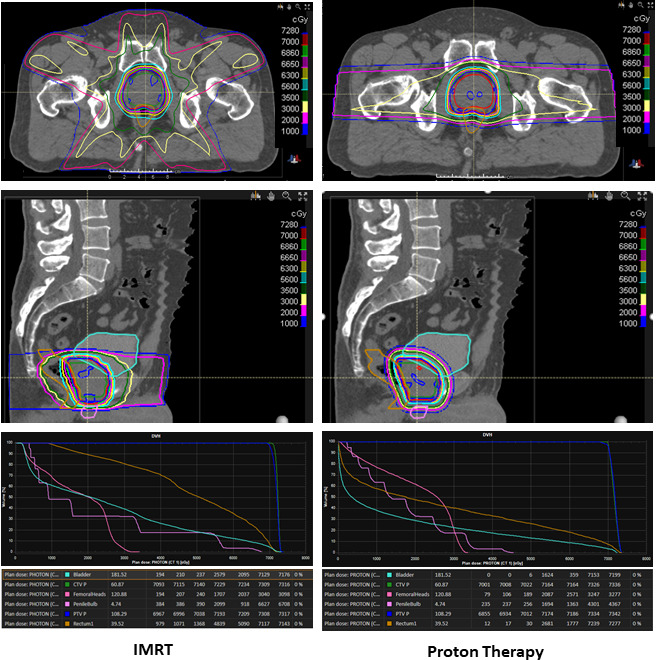
Comparison of intensity-modulated photon therapy (IMRT) versus double-scattered proton therapy for localized prostate cancer. The proton therapy plan reduces the radiation dose to the bladder, rectum, and penile bulb, but increases the dose to the femoral heads. Conformality with the prescription isodose line was similar between the two plans.

In the past, authors have shown that low to moderate doses of prostate radiation therapy can lead to toxicity. Vargas et al [5] established that the volume of rectum receiving 50 Gy (V50) when delivering photon-based radiation therapy to be associated with chronic grade 2^+^ rectal toxicity. Pederson et al [6] found that a rectum V40 of 40% or less reduced the risk for grade 2^+^ rectal toxicity after radiation therapy for prostate cancer delivered with IMRT. Low to moderate doses (V25-V40) delivered to the inferior rectum have also been associated with patient-reported GI outcomes after prostate radiation therapy [7]. Additionally, Yahya et al [8] found that urinary frequency after external-beam radiation therapy was associated with the amount of bladder volume receiving 41 Gy. These results, among others, have shown that the dosimetric improvements allowed by proton therapy may be clinically relevant.

Proton therapy has the potential to improve radiation dose homogeneity, especially within the PTV. Plan homogeneity is desirable with fractionated radiation therapy; a homogenous plan avoids delivering “hot spots,” which are areas of unintentionally elevated dose either within the target volume or in the surrounding organs at risk. Trofimov et al [2] showed that proton therapy reduced the maximum dose and the volume receiving more than 110% of the maximum dose when compared with IMRT among patients treated for prostate cancer.

Third, the risk for second cancer development among men treated with proton therapy for prostate cancer is likely lower than that for patients treated with IMRT. Yoon et al [4] found that IMRT plans generated a much higher integral dose than proton therapy plans, leading the authors to predict that IMRT was 5 times more likely to lead to secondary cancer development than proton therapy when treating prostate cancer. In a similar study, using second malignancy risk models following radiation therapy for prostate cancer, Fontenot et al [9] concluded that proton therapy could reduce the risk for events by 26% to 39% compared with IMRT. Chung et al [10] performed a retrospective cohort study of 558 patients treated with proton therapy and matched them to patients treated with photon radiation therapy. In each cohort, > 30% of patients had prostate cancer. Overall, at a median follow-up of 6.7 years, the risk for second malignancies was lower among patients treated with proton therapy than it was among those treated with photon radiation therapy (5.2% versus 7.5%; hazard ratio [HR], 0.52; *P* = .009). A study published by Chung et al [10] compared second malignancy rates for a broad range of patients with respect to diagnosis and age who were treated with 3D-conformal radiation therapy, IMRT, or protons, as inferred from diagnosis codes in a large US database. With a median follow-up of 5 years, the study showed a significantly lower rate of second cancers in patients treated with proton therapy compared with either IMRT or 3-dimensional conformal photon-based radiation techniques for all ages and disease sites, including prostate cancer. Although these previous 2 studies listed appear to show that proton therapy may reduce the risk for second malignancies, both were retrospective analyses and may suffer from biases from not accounting for risk factors, including demographic and socioeconomic circumstances, which can affect the risk for cancer.

## Potential Disadvantages of Proton Therapy

Depending on techniques compared, there may be disadvantages in dose distribution with proton therapy compared with IMRT. For example, Trofimov et al [2] found that IMRT provided better conformality of the high-dose volume to the target than double-scattered proton therapy did. The average conformality index was 2.73 with IMRT and 3.11 with the double-scattered proton therapy plans (*P* = .004). Similarly, in a comparison of IMRT to double-scattered proton therapy, Underwood et al [11] found that IMRT provided better high-dose conformality than proton therapy did when evaluating prescription isodose lines. In addition, IMRT provided lower volumes of both rectum- and bladder-receiving doses in the range of V50 to V70 compared with double-scattered proton therapy. This shortfall of double-scattered proton therapy can be eliminated by using IMPT, which is a more refined delivery method of proton particles using PBS. Trofimov et al [2] found that IMPT provided a better conformality index than IMRT did when delivering high-dose radiation therapy to the prostate.

Some have postulated there are greater uncertainties in the radiobiologic effectiveness and range of the proton beam that could be disadvantageous for proton therapy compared with photon therapy. The magnitude of cellular damage may be heterogeneous across a proton beam profile, complicating predictions of tumor response and toxicity. Although most clinical centers assume that proton therapy has a constant RBE of 1.1, several investigators have found that the RBE varies depending on beam angles and beam location [12, 13]. Preclinical data suggest that the RBE is > 1.1 at the distal edge of the spread-out Bragg peak [12, 13]. These variations, if ignored, could potentially place patients at greater risk for toxicity than they would have with photon radiation. For example, if a proton beam stopped on or within the anterior wall of the rectum, the rectal wall injury might be more than that predicted by standard dose-volume histogram parameters. Conversely, a variable RBE, if leveraged, could potentially aid in tumor control if areas of greater RBE are focused on the gross tumor volume. Any differential negative effects related to these potential uncertainties would need to be weighed against the significant reduction in overall integral dose with proton therapy.

## Current Indications for Proton Therapy in the Management of Prostate Cancer

Proton therapy is indicated in several clinical scenarios in the management of patients with prostate cancer in light of its potential to reduce the risk for acute and late toxicities related to dosimetric advantages compared with photon-based radiation therapy. The indications for proton therapy are listed in **Table 1** and are summarized below.

**Table 1.** Comparisons of and indications for intensity-modulated radiotherapy–volumetric modulated arc radiotherapy (IMRT-VMAT), double-scattering proton therapy (DSPT), and intensity-modulated proton therapy (IMPT).

**Technique**	**Advantages**	**Disadvantages**	**Clinical scenarios indicated**
IMRT-VMAT	High conformality of the prescription isodose lines around the target; robust and consistent for patient anatomy and patient motion	Increased dose to surrounding organs at risk in the low- to medium-dose range (0–50 GyRBE); potentially long beam-exposure times requiring intrafraction image guidance and position corrections	Localized prostate cancer; salvage or adjuvant prostate bed radiation therapy; elective pelvic node irradiation
DSPT	Reduced dose to surrounding organs at risk within the low- to medium-dose range (0–50 GyRBE); fast delivery times for targets without complex shapes; smoothing and smearing of dose with a compensator allows for robust proton therapy delivery for patient anatomy and motion	Low conformality of prescription isodose lines for complex targets; long treatment times for targets with complex shapes	Localized prostate cancer; salvage or adjuvant prostate bed radiation therapy
IMPT	High conformality with prescription isodose lines; reduced dose to surrounding organs at risk within the low- to medium-dose range (0–50 GyRBE)	Plan robustness for patient motion and anatomy can be challenging for targets with increased motion or significant changes in density or long beam delivery times; potentially long delivery times depending on spot scanning speed	Localized prostate cancer; salvage or adjuvant prostate bed radiation therapy; elective pelvic node radiation therapy; high-dose pelvic node radiation therapy delivered for patients with pelvic node adenopathy

### Localized Prostate Cancer

Several prospective cohort studies have documented the safety and efficacy of proton therapy in the management of localized prostate cancer. For example, results from a retrospective review of 1255 patients with localized prostate cancer were published by Slater et al [14]. Patients were treated with double-scattered proton therapy to 74 GyRBE at 2 GyRBE per fraction. At a median follow-up of 62 months, the 5-year biochemical relapse-free survival rate was 75% overall but was 90% for patients with an initial prostate-specific antigen level ≤ 4. The risk for late grade 3 GI and GU toxicity was < 1% for each.

Three clinical trials [15] of standard fractionated proton therapy conducted at the University of Florida in 212 patients demonstrated excellent 5-year biochemical control rates of 99% for low-risk, 99% for intermediate-risk, and 76% for high-risk disease with grade 3 GI and GU complications rates of < 1% and 2.9%, respectively, confirming the earlier excellent outcomes of Slater et al [14].

Bryant et al [16] published results from a larger unselected population of men treated at the University of Florida on a prospective outcome tracking protocol, which included 1327 men with localized prostate cancer. Patients were treated with 78 GyRBE at 2 GyRBE per fraction. Similar to the findings of the prospective clinical trials, the 5-year biochemical control rates were 99%, 94%, and 74%, respectively, for patients treated for low-, intermediate-, and high-risk prostate cancer. The risks for late grade 3^+^ GI and GU toxicity were 0.6% and 2.9%.

Takagi et al [17] published results of a retrospective study of 1375 patients treated with proton therapy for localized prostate cancer at the Proton Therapy Center at Sapporo Teishinkai Hospital in Japan. Patients were treated with 74 GyRBE at 2 GyRBE per fraction. The median follow-up was 70 months, and the 5-year rates for freedom from biochemical control were 99%, 91%, 86%, and 66% for patients with low-, intermediate-, high-, and very-high-risk prostate cancer, respectively. The cumulative grade 2^+^ toxicity rates were very low at 3.9% for GI and 2.0% for GU toxicity.

Iwata et al [18] published a multi-institutional retrospective series of 1291 patients treated with proton therapy in Japan with patients treated to a median dose of 74 GyRBE at 2 GyRBE per fraction. At 5 years, the biochemical control rates were 97%, 91%, and 83% for patients with low-, intermediate- and high-risk prostate cancer, respectively. The risks for grade 3^+^ GI and GU toxicities were 0.5% and 0.3%, respectively.

Makishima et al [19] published results of 111 patients with intermediate-risk prostate cancer treated with proton therapy at the Proton Medical Research Center at the University of Tskuba in Japan. Patients were treated with 78 GyRBE in 39 fractions using double-scattered techniques. At a median follow-up of 55 months, the rate of 5-year freedom from biochemical failure was 99%. Late grade-2 GI toxicity occurred in 4.3% of patients, and the late-grade 2^+^ GU toxicity rate was 5.8%. A single grade 3 toxicity occurred in 1 patient with noninfectious cystitis.

Overall, with follow-up extending out to 5 years, tumor control and toxicity outcomes appear to be at least on par with outcomes after delivery of IMRT. Additionally, outcomes appear to be consistent across multiple proton medical centers that span various regions, countries, decades, and patient ethnicities.

### Delivery of Hypofractionated Radiation Therapy

Although standard fractionation in prostate cancer is defined as doses of 76 to 80 Gy delivered in daily fractions of 1.8 to 2.0 Gy, moderately hypofractionated doses of 2.4 to 3.1 Gy have been used in an effort to make prostate cancer radiotherapy more convenient and less expensive for patients and insurers. In general, evidence has supported its noninferiority to conventional fractionation using photon-based radiation therapy in the management of low- to intermediate-risk prostate cancer [20–23], although 2 studies have shown a nonsignificant increase in late complications [20, 21]. In addition, a few studies have documented the safety and efficacy of moderate hypofractionation with proton therapy.

Henderson et al [24] reported outcomes for 215 men treated with proton therapy on a prospective trial. Patients with low- and intermediate-risk disease were treated to 70 GyRBE and 72.5 GyRBE, respectively, using 2.5 GyRBE per fraction. With a median follow-up of 5.4 years, biochemical control rates were 98.3% and 92.7% for patients with low- and intermediate-risk disease. Furthermore, the risks for grade 3 GI or GU toxicity were 0.5% and 1.7%, respectively.

Nakijima et al [25] published a retrospective comparison of acute toxicities after delivery of proton therapy for prostate cancer using conventional fractionation to 74 to 78 GyRBE at 2 GyRBE per fraction versus 60 to 63 GyRBE at 3 GyRBE per fraction. On univariate analysis, the investigators found that significantly less-acute GU toxicity was associated with hypofractionation (15% versus 5.9%; *P* < .001).

Finally, Grewal et al [26] reported 4-year results for patients with localized prostate cancer treated with 70 GyRBE of proton therapy in 28 fractions. The median follow-up was 49 months, and the 4-year biochemical relapse-free survival rates were 94.4%, 92.5%, and 93.8% for patients with low risk, favorable intermediate risk, and unfavorable intermediate risk, respectively. The rate of cumulative grade 2^+^ GU toxicity was 12.5%, and grade 2^+^ GI toxicity was 7.6%.

Although there is no randomized evidence proving the noninferiority of moderately hypofractionated proton therapy compared with standard fractionated proton therapy, published outcomes with proton therapy appear at least equivalent to hypofractionated IMRT. As a result, many proton centers are delivering hypofractionated proton therapy routinely. Still, the omission of proton therapy from the American Society for Radiation Oncology consensus statement as an appropriate modality for delivering hypofractionated radiotherapy indicates that more evidence is needed [27]. Consequently, there is an ongoing randomized comparison between standard and moderately hypofractionated proton therapy embedded in the Patient-Centered Outcomes Research Institute–funded COMPPARE study discussed below [28].

### Delivery of Stereotactic Body Radiation Therapy

Stereotactic body radiation therapy (SBRT) refers to the delivery of 5 fractions of 7 to 10 GyRBE. Because of its superior dosimetric sparing of organs at risk, proton therapy may prove beneficial in the delivery of SBRT for localized prostate cancer. By decreasing the number of fractions, the cost of a treatment course with proton therapy will decrease, and access to care as well as efficiency may improve for patients requiring radiation therapy. The benefits with proton therapy are manifold but, as with photon-based SBRT, comparative data are needed documenting its safety. A prospective comparative trial has been reported documenting similar early toxicity and tumor control for patients treated with conventionally fractionated proton therapy and SBRT for localized prostate cancer. Vargas et al [29] reported the results of a randomized proton therapy trial of 38 GyRBE over 5 fractions compared with 79.2 GyRBE over 44 fractions. These 2 doses were thought to be isoeffective in terms of healthy tissue toxicity. Only patients with low-risk prostate cancer and those with American Urological Association Symptom Index scores of ≤ 17 were included. At a median of 18 months, patients treated with SBRT had worse urinary QOL at 1 year, but by 18 months, no difference existed between the 2 groups. Furthermore, no differences were present in grade 2^+^ rectal or urinary toxicity at 18 months. As results from this trial and others mature, a role for SBRT delivery with proton therapy may emerge. For now, it should remain an active area of investigation.

### Delivery of Postprostatectomy Radiation Therapy

Postprostatectomy radiation therapy is indicated in the adjuvant or salvage setting after surgery, depending on the pathologic risk factors of recurrence after surgery. With conventional radiation therapy doses, biochemical disease control is achieved in only 50% to 60% of patients requiring radiation therapy as salvage treatment for prostate-specific antigen progression after prostatectomy [30]. Meanwhile, tumor control probability curves have consistently shown that doses > 70 Gy when delivering conventional fractionation may be needed to prevent recurrence or progression of prostate cancer [31]. Proton therapy may offer the potential for safe dose escalation in this setting. Deville et al [32] recently reported outcomes for patients treated with proton therapy to the prostate bed after prostatectomy. Patients were treated to 70.2 GyRBE at 1.8 GyRBE per fraction. The median follow-up was 55 months. The 5-year biochemical relapse-free survival rate was 56%. Toxicity was not reported. Additional studies will soon be reported evaluating the outcomes after adjuvant and salvage proton therapy for prostate cancer. These studies along with the findings of Deville et al [32] will further inform the medical community on the role of postprostatectomy proton therapy.

### Pelvic Node Radiation Therapy

When pelvic node radiation is indicated for patients with high-risk disease or pelvic adenopathy, proton therapy can provide dosimetric benefits by lowering the dose to the bowel in comparison to photon radiation. Chera et al [1] has shown that, in comparison with IMRT, proton therapy reduces the excess radiation delivered to the small bowel in elective pelvic node radiation therapy. Their findings suggest that this relationship may also hold true with dose escalation for the management of gross pelvic adenopathy. Patients who present with prostate cancer involving pelvic lymph nodes often pose a challenge to radiation oncologists. It is difficult to deliver the doses of radiation therapy necessary (60-70 Gy) to eliminate gross adenopathy without delivering an excess dose to the bowel and/or rectum, which can place the patient at high risk for bowel obstruction, high-grade diarrhea, and radiation proctitis. Proton therapy provides an opportunity for radiation dose-escalation to gross adenopathy within the pelvis while minimizing the dose to the bowel and rectal area. For patients who require radiation therapy to adenopathy within the para-aortic region, the benefit of proton therapy may help to reduce the dose to organs such as the duodenum, kidneys, and liver, which may reduce the risk of radiation-induced abdominal organ damage. Because of its improved dose conformality around complex targets, IMPT is preferred to double-scattered proton therapy in the delivery of nodal radiation, especially when high doses are delivered [33, 34].

## Comparative Studies of Proton Therapy versus Photon-Based Radiation Therapy

Gray et al [35] compared prospectively collected patient-reported QOL data for patients with localized prostate cancer treated with 3D conformal photon radiation, IMRT, or proton therapy. Data from 3 prospective cohort studies were used. Ninety-five patients were treated with proton therapy between 2004 and 2008 at Massachusetts General Hospital. The IMRT cohort included 153 men treated at 9 university hospitals between 2003 and 2006, comprising the Prostate Cancer Outcomes and Satisfaction with Treatment Quality Assessment (PROST-QA) Consortium. Finally, those patients treated with 3D conformal photon radiation therapy included 123 men treated at Harvard-associated hospitals between 1994 and 2000. After treatment, patient-reported outcomes were recorded and scored via Prostate Cancer Symptom Indices for the proton beam therapy and the Expanded Prostate Cancer Index Composite (EPIC) questionnaire. Radiation doses varied widely because those patients treated with proton therapy and IMRT were treated with doses between 74 and 82 Gy (or GyRBE), whereas patients treated with 3D conformal photon radiation were treated with 66 to 79 Gy. All doses were delivered at 1.8 to 2.0 Gy (GyRBE) per fraction. At 3 months after treatment, patient-reported QOL scores in the bowel/rectal, urinary irritative/obstructive, and incontinence domains were worse for patients treated with 3D conformal radiotherapy and IMRT than they were for those treated with proton therapy. At 2 years, no significant differences in patient-reported QOL were noted among the 3 treatment groups.

Hoppe et al [36] compared prospectively collected patient-reported QOL data for patients treated with proton therapy or photon radiation therapy for localized prostate cancer. The investigators analyzed prospective patient-reported QOL data from men with prostate cancer (n = 204) who received high-dose photon-based therapy through the PROST-QA study. Those patients were treated with IMRT and received doses between 75.6 and 79.2 Gy delivered at 1.8 to 2 Gy per fraction. Patients treated with proton therapy were from a single institution (n = 1226), and patient-reported outcomes were measured prospectively as well as after radiation therapy of 78 to 82 GyRBE delivered at 1.8 to 2 GyRBE per fraction. At 2 years of follow-up, there were no differences in EPIC bowel, urinary irritative/obstructive, or sexual summary scores between the 2 groups. On multivariate analysis, the patients treated with IMRT had significantly more “moderate” or “big problems” with rectal urgency and bowel frequency than did those treated with proton therapy.

Fang et al [37] performed a case-matched study comparing physician-reported toxicity for patients treated with IMRT or proton therapy for localized prostate cancer. Patients were treated at a single institution between 2010 and 2012 for localized prostate cancer. Patients were treated with 79.2 GyRBE at 1.8 GyRBE per fraction. Patients were matched based on risk group, age, and prior GU and GI comorbidities to minimize confounders. The median potential follow-up was 47 months for patients treated with IMRT and 29 months for patients treated with proton therapy. At 2 years of follow-up, there was no significant difference in physician-reported grade 2^+^ GU or GI toxicity between IMRT and proton therapy.

Finally, Pan et al [38] published a retrospective, matched, controlled study of patients with prostate cancer, comparing 693 treated with proton therapy and 3465 treated with IMRT. Patients were identified using medical claims data from the MarketScan Commercial Claims and Encounters database (IBM, Armonk, New York), and they were treated with radiation therapy between 2008 and 2015. Patients included received definitive radiation therapy with IMRT, proton therapy, or SBRT. Patients were matched using propensity scores based on clinical and sociodemographic factors. Patients were treated with similar radiation therapy doses, with nearly all received dose-escalated fractionated radiation therapy delivered at 1.8 to 2 GyRBE per fraction. Proton therapy appeared to lower the risk for urinary toxicity from 42% to 33% (*P* < .001) and erectile dysfunction from 28% to 21% (*P* < .001) when compared with those treated with IMRT. The risk for bowel toxicity was greater with proton therapy when compared with IMRT (10% versus 15%; *P* = .02).

Santos et al [39] published a retrospective review comparing proton therapy to IMRT in the postprostatectomy setting. Patients were treated with either salvage or adjuvant radiation therapy to a total dose of 66 to 70.2 Gy (or GyRBE) at 1.8 to 2.0 Gy (GyRBE) per fraction. Patients were treated using daily image guidance with a rectal balloon in place. The CTV volumes included the prostate bed only. Patients receiving proton therapy were treated with the PBS technique, whereas those treated with IMRT received 7- to 9-field coplanar static beams or volumetric-modulated arc beams. A matched case-cohort strategy was used to compare acute and late toxicities after treatment. At a median follow-up of 48 months and 46 months for patients treated with IMRT and proton therapy, respectively, there was no statistical difference in 5-year grade 2^+^ GU toxicity-free survival (61% versus 70%; *P* > .05). There was also no statistical difference in 5-year grade 1^+^ GI toxicity-free survival (74% versus 75%; *P* > .05).

Each retrospective comparison suffers from selection bias and a short follow-up. In addition, patient-reported QOL data were infrequently provided, which limits the validity of the results. Some of these studies compiled proton patients treated before 2005, when double-scattered proton therapy was delivered almost exclusively, and many patients were treated with a combination of IMRT and proton therapy using sequential plans. Patients treated at that time were also less likely to receive daily image guidance and were more likely to have large target volume expansions when compared with modern delivery of proton therapy. The combination of these factors could reduce the potential dosimetric advantages of proton therapy when compared with photon-based radiation therapy. It is expected that modern proton data with IMPT and more-advanced image-guided radiation therapy (IGRT), such as cone beam computed tomography and fiducial markers with or without rectal spacers, will better highlight proton therapy's potential when compared with modern IMRT. Because the dosimetric improvements possible with proton therapy reduce excess radiation in the low-to-moderate dose range, lower rates of erectile dysfunction, diarrhea, and bowel urgency are to be expected, which can be quantified with today's validated patient-reported QOL tools [40]. Additionally, the risk for some long-term toxicities continues to rise, even after 5 years of follow-up, including morbidity related to bladder function and the development of second malignancies. Consequently, studies with longer observation times are needed to accurately measure the effect of proton therapy.

## Technical Delivery of Proton Therapy

A minimum standard of treatment delivery must be followed to ensure the accuracy and effectiveness of proton therapy for prostate cancer. The goals of delivery should be to overcome 3 fundamental radiation therapy challenges: to minimize uncertainty regarding the precise location of the target during beam exposure, to maximize target coverage, and to minimize radiation exposure to organs at risk.

Because the prostate sits on the urogenital diaphragm and is not anchored to any bony structure, it is subject to interfraction displacement within the bony pelvis between the time of the simulation treatment planning with computed tomography imaging and the actual daily treatment. The more conformal the high-dose radiation volume is to the target, the more sensitive the treatment plan will be to small positional changes with both photon and proton therapy. Because the prostate may move independent of the pelvic bones, bony alignment will not guarantee accurate targeting of the prostate itself. To minimize prostate motion, patients should be simulated and treated when the bladder is full and the rectum is empty. Additionally, daily image-guided localization of either the prostate or implanted prostate fiducial markers should be used to precisely target the prostate immediately before treatment each day and to eliminate the problem of interfraction displacement [41]. Modern IGRT has incorporated the use of image guidance, including cone beam computed tomography and on-rail kilovoltage computed tomography, and these technologies should be used when available because they improve the accuracy of proton therapy.

The prostate is also subject to intrafraction position changes occurring during actual daily radiation exposures. Cine magnetic resonance imaging studies suggest that the prostate may become spontaneously displaced in the cephalad-caudad and anterior-posterior axes by ≤ 2 cm during a 10- to 20-minute time frame [42]. Variables affecting intrafraction motion include time required for radiation exposure, bladder filling, patient-specific peristalsis, rectal gas, rectal filling with either stool or instilled saline, and prostate stabilization techniques, such as the use of rectal balloons. The most common strategy for addressing uncertainty in position from intrafraction motion is expansion of the target based on anticipated potential movement, ie, a PTV that is commonly a 2- to 8-mm expansion of the prostate contour for treatment planning. Although some centers do not create a PTV volume, an expansion of target coverage around the CTV in some form is still needed to account for the risk of intrafraction motion. Expansions that are too small or too large can negatively affect clinical outcomes and introduce confounders that would be difficult to detect in future publications. Additionally, posttreatment position checks can be done to ensure that position deviations from the initial localization are adequately accounted for in the treatment planning process [43, 44]. Repeat imaging can be performed to ensure adequacy of the PTV margin.

Although intrafraction and interfraction variability must be accounted for with both proton- and photon-based treatment planning, there is a proton-specific uncertainty called “range uncertainty,” which refers to the effect on the proton beam range that the slight variations in prostate position may have on the composition of tissues (as well as on the proton stopping power) in the beam path. Range uncertainty is based on modeling studies and includes the addition of a margin to the proximal and distal edge of the target.

The instillation of rectal saline, insertion of rectal balloons, and injection of perirectal spacers to immobilize or displace parts of the rectal wall can improve many rectal dose-volume histogram parameters. These strategies have been shown to significantly affect not only dosimetric parameters but also clinically meaningful endpoints, including rectal toxicities [45]. The interfraction prostate position can also be affected by bladder filling and some centers perform pretreatment bladder scans with portable ultrasound to verify the bladder volume consistency. Consequently, when available, these technologies should be used for patients undergoing proton therapy for prostate cancer.

The 2 most common delivery methods for proton therapy for prostate cancer are PBS or double-scattered techniques. The PBS techniques allow for the modulation of beam intensity across a target to improve dose conformality over double-scattered proton therapy. At this time, no randomized prospective study has, to our knowledge, been performed that compares the techniques and shows whether one is superior in disease control, toxicity, or patient-reported outcomes. Retrospective outcomes have recently been published that may shed light on the comparison. Mishra et al [46] published a retrospective analysis of patient data from the Proton Collaborative Group registry. The study compared outcomes for patients treated for localized prostate cancer with PBS proton therapy versus those treated with uniform scanning or double-scattered proton therapy. A total of 1343 patients were included and treated to a mean radiation dose 79.2 GyRBE at 1.8 GyRBE per fraction. The median follow-up was 27 months for patients treated with double-scattered proton therapy or uniform scanning and 16 months for those treated with PBS proton therapy. Acute grade-2^+^ GU toxicity rates were higher for double-scattered proton therapy when compared with PBS (21.9% versus 15.1%, *P* < .01). Acute grade-2^+^ GI toxicity rates and late grade-2^+^ GI and GU toxicity rates did not differ between the 2 cohorts. This study suggests a potential improvement in toxicity rates with PBS but suffers from a potential confounding bias in that radiation plan dosimetry correlates were not accounted for by the study authors, which may have influenced the results. The median follow-up was also shorter than ideal given that late toxicity rates were the focus of the study [46].

## Cost-Effectiveness Analysis

Several authors have attempted to quantify the cost effectiveness of proton therapy in the management of prostate cancer by cost per quality-adjusted life year (QALY). The lack of long-term comparative outcome data or a defined cost associated with proton therapy limits the validity of those assessments. Konski et al [47] published results from a Markov model comparing proton therapy to IMRT for the definitive management of prostate cancer. The authors assumed delivery of dose-escalated proton therapy at 10 Gy higher than that of IMRT, which would lead to a 10% improvement in the 5-year freedom from biochemical failure. In terms of actual clinical outcomes, equivalent disease control rates have been observed with proton therapy treatment regimens employing 25% fewer treatment fractions [16, 48] and 10% to 15% improvements in 5-year freedom from biochemical failure rates with an equivalent number of treatment fractions as that used for IMRT [21, 24]. Proton therapy was assumed to yield a similar toxicity profile to that of IMRT, despite the increased dose, which may or may not be a valid assumption [36]. The cost of proton therapy was estimated from ambulatory payment classification rates from 2005, which took into account the cost for payers of each treatment and also the costs associated with prostate cancer recurrence. At 15 years, using a cost-effectiveness standard of US $50 000/QALY, the investigators found that proton therapy was not cost effective for a proposed 60- or 70-year-old man with prostate cancer.

Pan et al [38] published a retrospective comparison of proton therapy and IMRT using data from the MarketScan Commercial Claims and Encounters database of patients treated for definitive prostate cancer between 2005 and 2015. The mean costs to payers associated with proton therapy, IMRT, and SBRT were US $115 501, US $59 012, and US $49 504, respectively. At 2 years, proton therapy resulted in lower rates of GU toxicity and erectile dysfunction, but higher rates of grade-2 GI toxicity. Proton therapy was found to have a lower mean complication cost when compared with IMRT but a higher mean total health care cost. Unfortunately, estimates of freedom from clinical failure or biochemical control were not provided, and cost effectiveness was not estimated with a standard cost-utility model.

In 2008, the Institute for Clinical and Economic Review [49] provided a cost-effectiveness analysis of proton therapy in the management of prostate cancer. The authors performed a systematic review of the literature to determine the estimated risk for complications and the potential for disease control with proton therapy when compared with IMRT and brachytherapy. Only one retrospective study on proton therapy was used in this analysis, which limits the usefulness of the results. The therapies were assumed to yield similar disease control rates, and proton therapy was thought to have lower acute- and late-toxicity rates based on the literature review. Costs were estimated from Medicare payments in 2007 using procedural codes, ambulatory payment codes, and relative value units. Proton therapy was assumed to cost US $48 493 for an 8-week regimen compared with US $19 760 for IMRT and US $10 024 for brachytherapy. Costs associated with the management of toxicities, and patient time was also estimated. The authors concluded that brachytherapy was the most cost-effective treatment for prostate cancer. They found that IMRT was more cost effective than proton therapy, and when sensitivity analyses were performed on patients 58 years or younger, the results remained similar.

Although several cost effectiveness analyses have been published, each has presented results that may have limitations for a variety of reasons. Investigators who have attempted such cost analyses are forced to make assumptions about a number of important parameters that affect long-term efficacy and safety outcomes and may lead to inaccurate conclusions [50]. Additionally, when comparing toxicity profiles, investigators often use photon-based predictors from normal tissue complication probability models, which are now known to be inaccurate in predicting outcomes with proton therapy [51]. Additionally, when developing cost-effectiveness estimates, most investigators assume higher costs for payers with proton therapy; however, because many radiation therapy centers have negotiated price parity among the modalities or provide proton therapy at a cost similar to IMRT, any cost-effectiveness comparisons are inapplicable to many of the nation's busiest proton centers. Critics of the use of cost per QALYs cite its empirical nature. Moreover, QALY is not designed for valuing health improvements but, rather, for valuing health costs to payers. Assuming a fixed number of dollars per QALY may be too simplistic for some models, and the heterogeneity of estimates that quantify willingness to pay for QALY is well known [52]. Furthermore, the results of the European Union–funded European Consortium in Healthcare Outcomes and Cost-Benefit Research experiment have shown that health assessments expressed in number of QALYs or cost per QALY are inconsistent and can lead to divergent results because the underlying assumptions of the QALY model are not validated [53]. In the era of personalized medicine and breakthrough innovations, the cost per QALY model has been found to be imprecise.

## Current Trials

As noted in **Table 2**, several prospective trials are currently underway in the United States, which will provide greater clarity on the role of proton therapy in the management of prostate cancer. The results of 2 major prospective multi-institutional trials are anticipated in a few years. The results of each trial will have overarching ramifications for the future of proton therapy for prostate cancer but if the trials provide clarity on the utility of proton therapy, each will do so in very different ways. The Prostate Advanced Radiation Technologies Investigating Quality of Life (PARTIQoL) trial [55] is a multi-institutional, randomized trial comparing proton therapy to IMRT in the definitive management of localized prostate cancer. Patients with low- or intermediate-risk prostate cancer will be treated with fractionated radiation therapy to 79.2 GyRBE at 1.8 GyRBE per fraction or 70 GyRBE at 2.5 GyRBE per fraction. Participants will be randomized to proton therapy or IMRT, and each plan will be delivered per rigid guidelines with central review. No androgen-deprivation therapy is allowed. Patients may be treated with either double-scattered proton therapy or pencil beam scanning and SpaceOAR Hydrogel (Boston Scientific, Marlborough, Massachusetts) insertion is allowed. The study hypothesis is that proton therapy will improve patient-reported QOL. The primary end point of the study will be the EPIC bowel summary score at 24 months of follow-up. Secondary end points will include urinary and erectile function. Because of this trial's rigorous enrollment criteria, randomization, and treatment guidelines, internal validity should be a relative strength, and the results will carry weight for the specific types of patients enrolled and the results related to patient-reported QOL. Because of a lack of statistical power, the study is not expected provide any additional information regarding tumor control, physician-reported toxicity, or the influence of modifiers, such as rectal spacers, pencil-beam scanning, patient ethnicity, and comorbidities.

**Table 2.** Clinical trials using proton therapy in prostate cancer.

**Status**	**Title**	**ClinicalTrials.gov identifier**	**Principal center**
Phase 3 trials			
Recruiting	A phase III prospective randomized trial of standard-fractionation vs. hypo-fractionation with proton radiation therapy for low risk adenocarcinoma of the prostate	NCT01230866 [54]	Mayo Clinic in Arizona (Phoenix)
Recruiting	Proton therapy vs. IMRT for low or intermediate risk prostate cancer (PARTIQoL)	NCT01617161 [55]	Massachusetts General Hospital (Boston)
Recruiting	Hypo-fractionated radiation therapy with or without androgen suppression for intermediate risk prostate cancer	NCT01492972 [56]	Mayo Clinic in Arizona (Phoenix)
Recruiting	Prostate cancer patients treated with alternative radiation oncology strategies (PAROS)	NCT04083937 [57]	University Hospital Heidelberg (Heidelberg, Germany)
Phase 2 trials			
Recruiting	A phase II trial of hypofractionated radiation therapy for prostate cancer with high risk features after radical prostatectomy	NCT03570827 [58]	Mayo Clinic in Arizona (Phoenix)
Recruiting	A phase II trial of proton radiation therapy or intensity-modulated radiation therapy using mild hypofractionation for low- and intermediate-risk adenocarcinoma of the prostate	NCT01352429 [59]	University of Pennsylvania (Philadelphia)
Recruiting	Phase II trial of hypofractionated proton beam therapy in men with localized prostate adenocarcinoma	NCT01950351 [60]	MD Anderson Cancer Center (Houston, Texas)
Recruiting	A phase II study of dose-escalated proton-based radiation therapy delivered with a simultaneous integrated boost (SIB) to intraprostatic tumors (IPT) visible on pretreatment magnetic resonance image	NCT03624660 [61]	University of Florida Health Proton Therapy Institute (Jacksonville)
Recruiting	A prospective comparative study of outcomes with proton and photon radiation in prostate cancer (COMPPARE)	NCT03561220 [28]	University of Florida Health Proton Therapy Institute (Jacksonville)
Recruiting	Postoperative or salvage radiotherapy for node negative prostate cancer following radical prostatectomy	NCT00969111 [62]	University of Florida Health Proton Therapy Institute (Jacksonville)
Recruiting	Proton-based stereotactic ablative body radiotherapy (SABR) for select patients with clinically localized prostate cancer	NCT03159676 [63]	Mayo Clinic in Rochester (Rochester, Minnesota)
Recruiting	Phase I–II trial of hypofractionated conformal proton beam radiation therapy for favorable-risk prostate cancer	NCT00831623 [64]	Loma Linda University (Loma Linda, California)
Recruiting	A phase II study of hypofractionated image guided proton therapy for low and intermediate risk prostate cancer	NCT02040610 [65]	Provision CARES Proton Therapy Center (Knoxville, Tennessee)

A second trial, the COMPPARE trial has recently begun accrual in the hopes of contributing to our understanding of the efficacy of proton therapy in comparison with photon therapy. Participants with localized low-, intermediate-, or high-risk prostate cancer are enrolled into 1 of 2 study cohorts: a proton cohort or an IMRT cohort. Patients can be treated with proton therapy or IMRT depending on patient, physician, and institution discretion regarding standard-of-care strategies: standard or moderate hypofractionation, with or without androgen-deprivation according to National Comprehensive Cancer Network guidelines, and with or without pelvic-node irradiation according to National Comprehensive Cancer Network guidelines and standard nomograms. Within the proton cohort, there is a nested randomized trial comparing 2 radiation fractionation schemes: 60 GyRBE at 3 GyRBE per fraction and 78 GyRBE at 2 GyRBE per fraction. The study is considered pragmatic because the entry criteria are broad, and radiation treatment guidelines are recommendations rather than restrictions. A total of 3000 patients will be enrolled and, with these relatively large patient numbers, the investigators anticipate being able to answer several questions regarding the relative benefits of proton therapy in the management of prostate cancer by analyzing patient-reported QOL, tumor control, and physician-reported toxicity. The study investigators also aim to determine whether moderate hypofractionation is noninferior to conventional fractionation for prostate cancer and how the type of radiation therapy interacts with the clinical outcomes. This trial has the potential to provide valuable and more-broadly applicable data for most patients with prostate cancer treated with external radiation, and its results will complement those of the PARTIQoL study.

The prostate bed irradiation with alternative radio-oncological approaches (PAROS) [57] trial is a randomized trial that includes patients with prostate cancer in need of adjuvant radiation therapy after prostatectomy. Patients will be randomized to fractionated photon-based radiation therapy (70 Gy at 2 Gy per fraction), hypofractionated proton therapy (57 GyRBE at 3.0 GyRBE per fraction), or hypofractionated photon-based radiation therapy (57 Gy at 3.0 Gy per fraction). The primary end point of the study is bowel-related QOL, and the hypothesis is that proton therapy will improve bowel-related QOL compared with photon-based radiation therapy. An important secondary end point will be to determine whether hypofractionated radiation therapy is noninferior to fractionated radiation therapy for bowel QOL. This study will have a major effect not only on the method of radiation therapy delivery but also on the length of radiation therapy for patients in need of radiation therapy after surgery.

## Conclusions

As an established and effective treatment for patients with prostate cancer, proton therapy reduces the excess radiation delivered to healthy tissues surrounding the prostate when compared with photon-based radiation therapy. Several prospective and retrospective studies have been published documenting the safety and efficacy of proton therapy in the management of prostate cancer and some long-term follow-up data are available and are accumulating. Consequently, proton therapy should not be considered experimental in the management of prostate cancer. It is efficacious when delivered to patients with localized prostate cancer or when delivered postoperatively. It also can be delivered safely to patients requiring pelvic nodal radiation for high-risk or node-positive disease. Although the radiobiologic uncertainties of proton therapy are not fully understood, as our knowledge grows, the advantages and limitations of proton therapy will become clearer. Soon, the results of multiple prospective studies will also contribute to our understanding of the comparative safety and efficacy of proton therapy in the management of prostate cancer. Because the costs associated with proton therapy continue to decrease, further evidence of proton therapy as an effective platform for hypofractionation may prove it to be a cost-effective treatment modality in the management of prostate cancer.
